# Paclitaxel Regulates TRPA1 Function and Expression Through PKA and PKC

**DOI:** 10.1007/s11064-022-03748-0

**Published:** 2022-09-13

**Authors:** Julio C. Sánchez, Laura V. Muñoz, María-Leonor Galindo-Márquez, Aníbal Valencia-Vásquez, Andrés M. García

**Affiliations:** Faculty of Health Sciences, Universidad Tecnológica de Pereira, AA 97, La Julita, 660003 Pereira, Colombia

**Keywords:** Transient receptor potential A1 channel, Calcium transients, Chemotherapy-induced peripheral neuropathy, Neuroblastoma cells, Neurotoxicity, Paclitaxel

## Abstract

Paclitaxel (PTX) is a frequently used anticancer drug that causes peripheral neuropathy. Transient receptor potential ankyrin 1 (TRPA1), a plasma membrane calcium channel, has been associated with PTX toxicity and with other chemotherapy agents such as oxaliplatin and vincristine. However, the effect of PTX on the functional expression and calcium currents of TRPA1 has not been determined. The present study shows the effect of PTX on TRPA1 activity in a neuronal cell line (SH-SY5Y). The effect of PTX on the expression of TRPA1 was assessed through quantitative PCR and Western blot analyses to determine the relative mRNA and protein expression levels. To assess the effect on calcium flux and currents, cells were exposed to PTX; simultaneously, a specific agonist and antagonist of TRPA1 were added to evaluate the differential response in exposed versus control cells. To assess the effect of PKA, PKC and PI3K on PTX-induced TRPA1 increased activity, selective inhibitors were added to these previous experiments. PTX increased the mRNA and protein expression of TRPA1 as well as the TRPA1-mediated Ca^2+^ currents and intracellular Ca^2+^ concentrations. This effect was dependent on AITC (a selective specific agonist) and was abolished with HC-030031 (a selective specific antagonist). The inhibition of PKA and PKC reduced the effect of PTX on the functional expression of TRPA1, whereas the inhibition of PI3K had no effects. PTX-induced neuropathy involves TRPA1 activity through an increase in functional expression and is regulated by PKA and PKC signaling. These findings support the role of the TRPA1 channel in the mechanisms altered by PTX, which can be involved in the process that lead to chemotherapy-induced neuropathy.

**Supplementary Information:**

The online version contains supplementary material available at 10.1007/s11064-022-03748-0.

## Introduction

Paclitaxel (PTX) is an antineoplastic and cytotoxic agent that exhibits high efficiency in treating ovarian, breast and lung cancers [1] by affecting the polymerization of microtubules in the cytoplasm and, thus, axonal transport [2]. PTX is associated with nervous system disorders including neuropathic pain, peripheral neuropathy [3, 4], acute pain syndrome [5, 6] and cognitive impairment [7]; these side effects are mostly dose-dependent, have a prevalence between 44 and 98% [8, 9] and cause frustration and disability in patients [10]. It is well known that the mechanism by which PTX causes peripheral neurotoxicity is different from its therapeutic action [2]. Although the exact underlying mechanisms are not well known, processes such as mitochondrial dysfunction, oxidative stress, inflammation and transient receptor potential (TRP) channel hyperactivity have been reported to be involved [11]. Furthermore, PTX-induced central neurotoxicity is associated with microtubule dysfunction, impaired neurogenesis, neuroinflammation, and apoptosis [11]. Altered intracellular Ca^2+^ signaling is a common factor among the molecular mechanisms involved in neurotoxicity [12].

Different preclinical models have shown the involvement of transient receptor potential ankyrin 1 (TRPA1) channels in cancer pain [13, 14] and chemotherapy-induced neuropathic pain [15–17]. TRPA1 is a homo or heterotetrameric nonselective cation channel that is mainly permeable to calcium (Ca^2+^) [18]. TRPA1 is mainly expressed in small-diameter C or A delta-fibers of sensory neurons, such as dorsal root ganglion (DRG) [19] and trigeminal neurons [20], and it sometimes coexpressed with TRPV1 channels [21]. Additionally, TRPA1 expression and function have been demonstrated in the rodent spinal dorsal horn and somatosensory cortex [22, 23]. The primary function of TRPA1 is to be a nociceptive sensor of various chemical compounds, which are also involved in thermosensation and mechanotransduction [24, 25]. The TRPA1 channel is activated by allyl isothiocyanate (AITC) [26] and inhibited by selective antagonists such as HC-030031 (HC-030) [17] and A-967079 [27]. Given its sensitivity to various compounds and stimuli, TRPA1 is involved in different physiological responses such as pain [17], itching [28], and inflammation [29].

Several studies have demonstrated the participation of TRPA1 channels in chemotherapy-induced peripheral neurotoxicity (CIPN). Antagonism of TRPA1 produces an antinociceptive effect in rodent models of cancer pain [13, 14]. Moreover, activation of TRPA1 in cancer pain models increases production of hydrogen peroxide (H_2_O_2_), which maintains TRPA1 activation/sensitization and these H_2_O_2_ levels may be caused by an augmented activity of NADPH oxidase and superoxide dismutase as well [14]. Although it has been reported that ROS are associated with CIPN, a specific receptor gated by reactive compounds involved in cancer pain remains unknown [30]. Early treatment with TRPA1 inhibitors prevents the mediation of CIPN mediated by oxidative stress byproducts, including those generated by PTX [17].

PTX treatment generates activation of Toll-like receptor 4 (TLR4) [31–33], leading to the release of tumor necrosis factor-α (TNF- α) from satellite glial cells in the DRG, which increases the expression of TRPA1 channels in these neurons [31]. In contrast, chronic exposure of DRG cultures to PTX inhibits calcitonin gene-related peptide (CGRP) release mediated by TRPA1 agonists [34]. In the PTX-induced neuropathic pain model, TRPA1 channels are regulated by phosphorylation through proteinase-activated receptor 2 (PAR2) activation [15]. Inhibition of TRPA1 channels reduces cold and mechanical allodynia induced by PTX, which involves reactive oxygen species (ROS) formation [16]. Thus, ROS activate TRPA1 channels to increase the excitability of spinal dorsal horn neurons [35].

In the present study, we investigated the effects of PTX on TRPA1 channel expression and function in human neuroblastoma cells as a neuronal model for PTX-induced neurotoxicity.

## Methods

### Chemicals and Reagents

All chemicals and solutions were obtained from Sigma-Aldrich (St. Louis, MO, USA), unless otherwise stated. PTX, Fura-2-AM, AITC, HC-030, H89 and wortmannin were dissolved in DMSO as stock solutions (0.01–1 M). Chelerythrine was dissolved and stored in sterile water.

### Culture of Human Neuroblastoma Cells

SH-SY5Y cells were obtained from the European Collection of Authenticated Cell Cultures (ECACC), were cultured in DMEM/F12 medium (1:1, v/v; Gibco-Invitrogen) supplemented with 10% fetal bovine serum, 100 U/ml penicillin, and 100 µg/ml streptomycin. Cultured cells were maintained at 37 °C in a 95% humidified atmosphere with 5% CO2. The culture medium was changed every other day. The morphological characteristics were monitored to assure the differentiation of the cells. When cell confluence reached 80%, cells were detached by TripLE Express (1X) and seeded in 6-well and 96-well plates based on the experimental requirements. The cells were employed in the subsequent experiments without any additional treatment.

### Quantitative RT-PCR

For detection of the human TRPA1 gene, total RNA from cell cultures was extracted with an RNeasy Mini Kit based on the manufacturer's protocol and quantified using a Multiskan TM Microplate Spectrophotometer (Thermo Scientific). The expression was assessed using the respective probes for TRPA1 (Hs00175798_m1) and for β-actin (Hs01060665_g1). PCR was performed with the TaqMan® RNA-to-CT TM 1-Step Kit based on the manufacturer’s protocol. The following thermocycler protocol was applied: initial denaturation at − 50 °C (30 min) and 95 °C (15 min) followed by 40 cycles of 95 °C (15 s) and 60 °C (60 s). The fluorescence was obtained for each amplification cycle and the data were analyzed using the 2^−ΔΔCt^ method for the relative quantification of expression.

### Western Blotting

Cells were seeded in 6-well plates at a density of 2 × 10^6^ cells/well. After reaching 80% confluence, cells were washed with ice-cold PBS, harvested in Pierce™ RIPA Lysis Buffer (Thermo Scientific) and scraped off. The lysate was centrifuged at 14,000×*g* for 30 min at 4 °C. The supernatant was collected, and the protein concentration was determined using a Pierce™ BCA Protein Assay Kit (Thermo Scientific). Cell lysates containing 20 µg of protein were separated by SDS-PAGE followed by electrophoretic transfer onto PVDF membranes. Membranes were blocked in 5% fat free milk in PBS containing 0.05% Tween 20. Membranes were then incubated with the following primary antibodies overnight at 4 °C: anti-TRPA1 (NB110-40763SS Novus BIOLOGICALS) and anti-β-actin (MA1-140 Thermo Fisher Scientific). After incubation with anti-rabbit IgG, HRP-linked antibody (Cell Siganling, ref: 7074S) for 2 h at room temperature, the bands were visualized by a ChemiDoc™ MP system (Bio-Rad Laboratories, Inc, USA).

### Electrophysiological Recordings

The whole-cell patch clamp technique was used to record the membrane currents (voltage clamp) and membrane potential (current clamp) in cultured SH-SY5Y cells. Cells were placed in a recording chamber attached to an inverted microscope (Nikon, Tokyo, Japan). Patch pipettes (Clark PG150T glass, Harvard Apparatus Ltd, Edenbridge, Kent, UK) were pulled and polished (P-97, Sutter Instrument, Novato, CA, USA) to resistances of 5–10 MΩ. After a seal of resistance greater than 5 GΩ was obtained, membrane currents were recorded using an Axopatch 200B amplifier with a CV203BU headstage (Axon Instruments Inc., Union City, CA, USA). Voltage clamp signals were generated by a Digidata 1440A interface (Axon Instruments Inc.). Membrane currents were filtered at 2 kHz and digitized at a sampling rate of 10 kHz. The signals were acquired and analyzed using pCLAMP 10.0 (Axon Instruments Inc.). At the beginning of each experiment, the junction potential between the pipette solution and bath solution was electronically adjusted to zero. The macroscopic current values were normalized as pA/pF.

Single TRPA1 channel activity was investigated using inside-out patch clamp recordings [36]. Single channel unitary current (i) was determined from the best fit Gaussian distribution of amplitude histograms. Channel activity was analyzed using the following equation: *NP*_*o*_ = *I/i*, where I is the mean total current in a patch and i is the unitary current at this voltage. Where appropriate, open probability (P_o_) was calculated by normalizing NP_o_ to the total number of estimated active channels (N) in the patch. To increase accuracy in the measurement of P_o_, only patches containing fewer than three channels were used. All experiments were performed at room temperature (20 °C).

The standard external solution contained (mM) 30 NaCl, 4 KCl, 2 CaCl_2_, 1 MgCl_2_, 10 HEPES, 10 glucose and 180 mannitol with the pH adjusted to 7.4 at 25 °C using 1 M NaOH. The standard pipette solution contained (mM) 25 KCl, 5 K-gluconate, 5 NaCl, 4 CaCl_2_, 4 MgCl_2_, 10 HEPES, 5 glucose, 20 BAPTA and 180 mannitol with the pH adjusted to 7.1 at 25 °C using 1 M NaOH. The osmolarity of these solutions was adjusted to 290 mOsm/L using 5 M mannitol. The free Ca^2+^ concentration was estimated using ‘Maxchelator’ software (Dr C. Patton, Stanford University) and a value of 102 nM was derived for the intrapipette solution using this software. In the Na^+^-free solutions, Na^+^ was replaced with NMDG^+^ in equimolar concentrations. EGTA (1 mM) was added to the Ca^2+^-free solutions to chelate contaminant traces of this ion. For the Cl^−^-free intrapipette solutions, NaCl and KCl were replaced by Na-gluconate and K-gluconate, respectively. The osmolarities of all solutions were measured using an Advanced Model 3320 Micro-Osmometer (Advanced Instruments, Norwood MA, USA). The solutions were switched using the cFlow V2.x flow controller (Cell Microcontrols, Norfolk, VA, USA).

### Measurement of Intracellular Calcium Concentration Ca^2+^

Before exposure to PTX, AITC, and HC-030, cultured SH-SY5Y cells were loaded with Fura-2-AM (5 µmol/L) by incubation in HBS (mM, 145 NaCl, 5 KCl, 2 CaCl_2_, 15 HEPES and 10 glucose with the pH adjusted to 7.4 at 25 °C using 1 M NaOH) for 30 min at 20 °C, followed by 15 min at 37 °C. The cell suspension was then centrifuged, and cells were resuspended in the appropriate experimental medium before being transferred to a cuvette. Fluorometer measurements were made for 300 s at 37 °C with magnetic stirring (FP-6500 spectrophotometer, Jasco, Tokyo, Japan) during the measurements. The dye was alternately excited at 380 nm and 340 nm, and the fluorescence emission was measured at 510 nm. The 380 nm/340 nm signal ratio was calibrated before every experiment using a previous method [37]. Briefly, the fluorescence ratio was measured in HBS without CaCl_2_ and supplemented with EGTA (1 mmol/L) as well as in a 2 mmol/L Ca^2+^ HBS supplemented with ionomycin (300 nmol/L), a concentration of Ca^2+^ at which Fura-2 is saturated. Maximal and minimal ratios (R_max_ and R_min_) were obtained under these two conditions, and the [Ca^2+^]_i_ values were derived using the following equation:

$$\left[ {{\text{Ca}}^{2 + } } \right]_{i} = \, K_{d} \left( {\left[ {R - R_{min} } \right]/\left[ {R_{max} - R} \right]} \right)\left( {{\text{Sf}}_{2} /{\text{Sb}}_{2} } \right),$$

where K_d_ is the dissociation constant for Fura-2 (224 nmol/L), R is the experimentally measured ratio; Sf_2_ is the fluorescence measured at 380 nm under Ca^2+^-free conditions; and Sb_2_ is the fluorescence measured at 380 nm with saturating Ca^2+^ (2 mmol/L).

### Statistical Analysis

Data analysis was performed using the analysis tools available in pCLAMP 10.0 software and in GraphPad Prism 9.0. The results are shown as the mean ± standard error of the mean [38], where n is the number of cells tested. Each experimental observation was repeated in at least six different cells. An unpaired Student´s t-test analysis was performed, when it was suitable; otherwise, the corresponding nonparametric test was used. All statistical tests were performed with two-tailed tests and a p value < 0.05 was considered significant.

## Results

### Expression of TRPA1 and Effects of PTX on Protein Levels

**Fig. 1:**
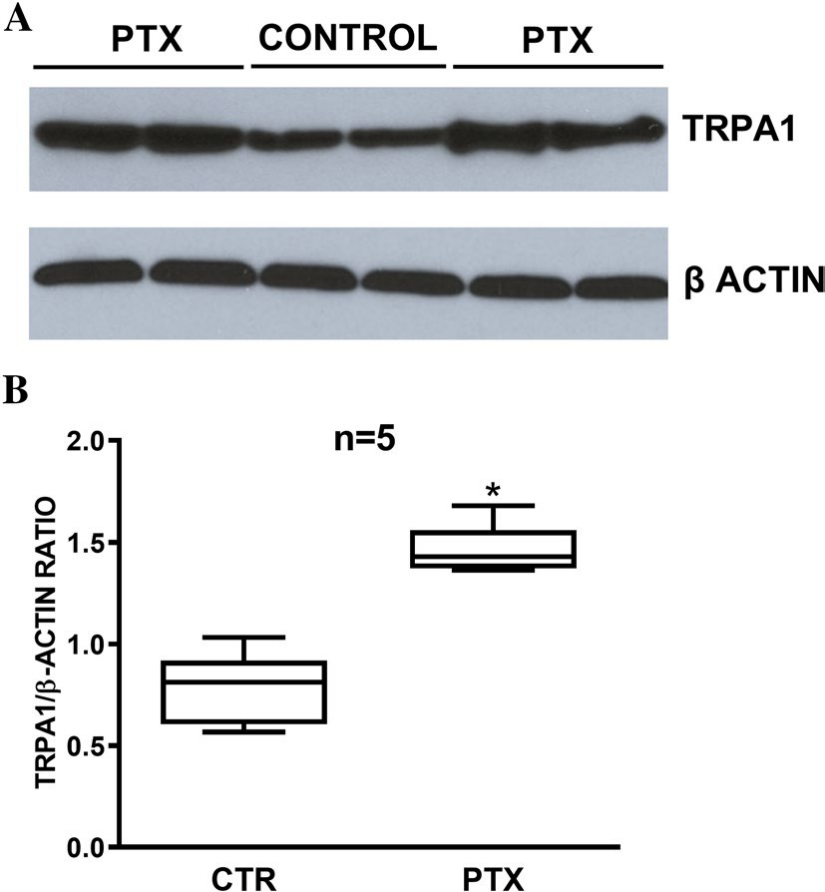
**A** Representative Western blot showing the expression of TRPA1 protein in SH-SY5Y cells under basal conditions and following a 6-h PTX treatment. Human β-actin was used as a control. Note the different protein levels in both conditions. **B** Comparative levels of TRPA1 expression in SH-SY5Y cells normalized to levels of β-actin expression (n = 5). *Denotes a significant increase (p = 0.0001) compared to the control

To determine the functional expression of the TRPA1 gene, qPCR and Western blotting (WB) experiments were performed in SH-SY5Y cells in the presence and absence of PTX (1 µM). In qPCR experiments, PTX significantly increased the relative expression of TRPA1 by 1.55-fold (n = 3, *p* = 0.028). In WB experiments, the expression of TRPA1 was confirmed by the identification of a band at 127 kDa, as expected for TRPA1 in other cell types, under basal conditions. The expression of β-actin was used as a control. After PTX pretreatment for 6 h, the TRPA1 protein levels significantly increased compared to the control group (Fig. 1).

### Effects of PTX on TRPA1 Currents

**Fig. 2:**
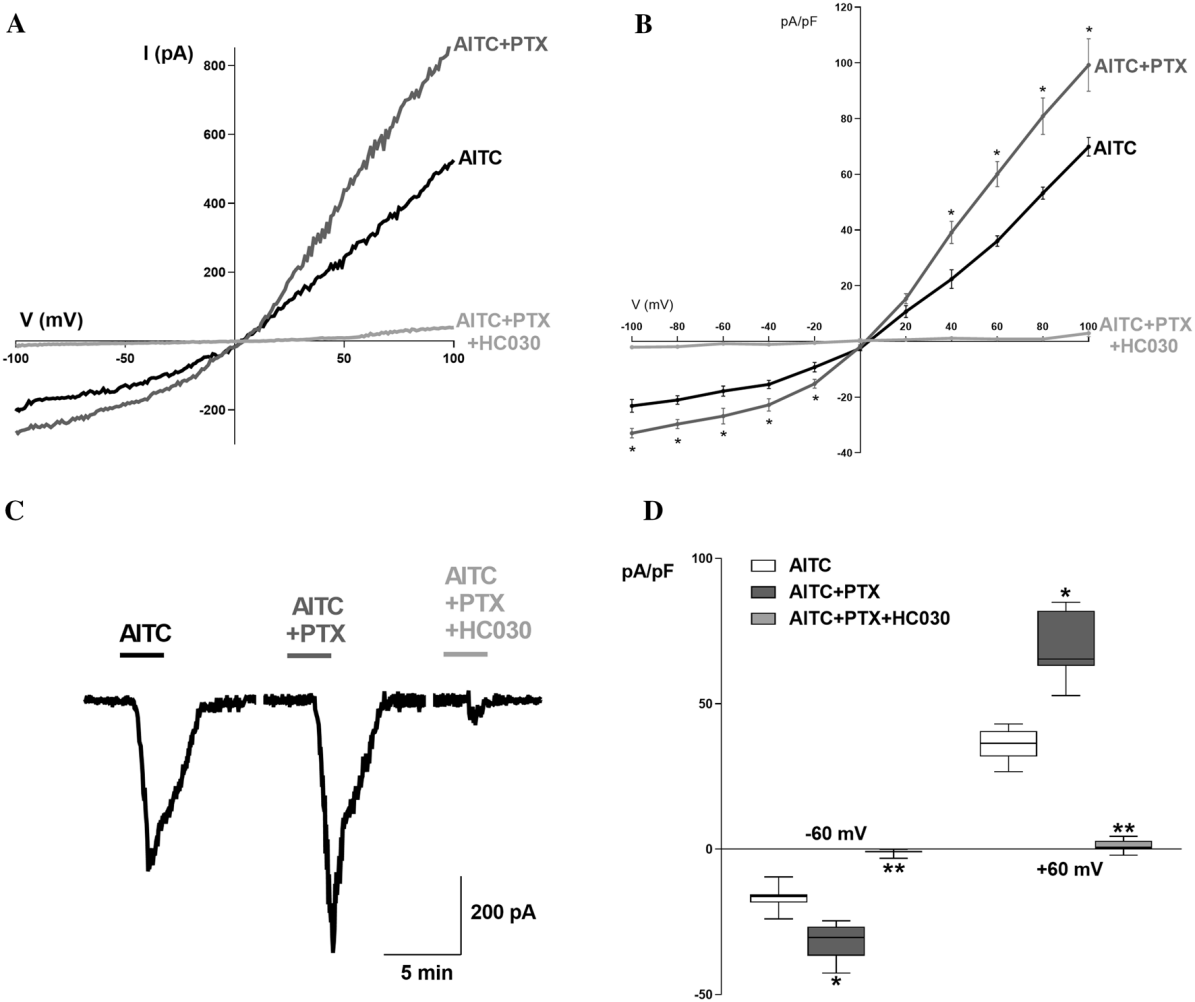
**A** Typical I-V recording of the current obtained in a SH-SY5Y cell, elicited by a ramp protocol from − 100 mV to + 100 mV and activated by AITC in control conditions and the presence of PTX and HC-030. **B** Typical I–V relationship of the AITC-induced current in SH-SY5Y cells in control conditions and the presence of PTX and HC-030. *Denotes significant differences. **C** Typical current (I) recording at − 60 mV in the presence of AITC, AITC + PTX and AITC + PTX + HC030, as indicated. **D** Comparison between the mean maximal normalized current recorded at − 60 mV and + 60 mV under the same conditions. n = 8 in all cases. *Denotes significance (p = 0.02)

Using the whole-cell patch clamp technique, a descending ramp protocol was applied, in which the membrane potential was stepped from a − 60 mV holding potential to + 100 mV for 100 ms and then ramped to − 100 mV over 2 s. Stimulation with AITC (300 µM), a potent TRPA1 agonist, elicited a predominantly inward current, which exhibited outward rectification. The current was decreased by the specific inhibitor HC-030031 (HC030, 50 µM), which confirmed that it was a TRPA1-mediated current (Fig. 2).

To assess the acute effect of PTX on the TRPA1 current, PTX (1 µM) was added to the extracellular solution before the ramp protocol in the presence of AITC and the current–voltage relationships were derived. After 1-min exposure, PTX increased the magnitudes of the inward and outward current densities compared to control cells. The effect of PTX on the AITC-induced current was reversed when it was washed out from the extracellular solution. When cells were treated with PTX only, this drug failed to elicit any electrophysiological change indicating that the effect of PTX occurred only on the open channel.

To evaluate the role of protein kinase A (PKA), protein kinase C (PKC) and phosphoinositide 3-kinase (PI3K), a specific inhibitor of each kinase was added to the extracellular solution after the administration of PTX and activation of AITC-induced current. The inhibition of PKA (10 µM H89, a selective PKA inhibitor) and PKC (10 µM chelerythrine, a selective PKC inhibitor), decreased the magnitudes of the inward and outward current densities compared to PTX-stimulated cells, and the inhibition of PI3K (1 µM wortmannin, a selective inhibitor at this concentration) did not elicit changes in the current densities (Fig. 5A and B).

**Fig. 3:**
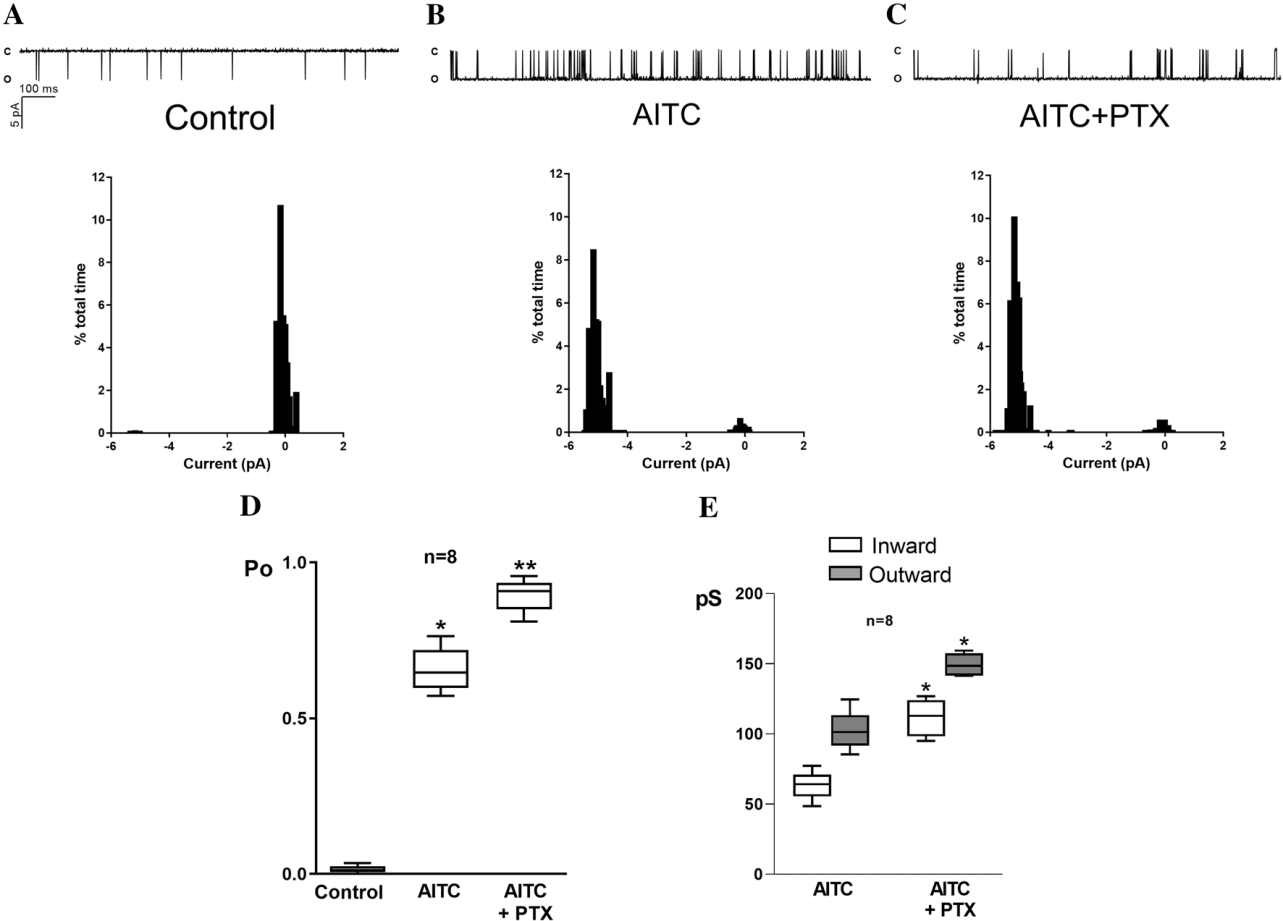
Recordings of single-channel activity and corresponding amplitude histogram of single-channel currents measured at − 60 mV in inside-out patches from SH- SY5Y cells under basal conditions (**A**), in the presence of AITC (**B**), and in the presence of AITC and PTX (**C**). **D** Comparison of the mean open probability (Po) in 8 different patches in the conditions described in **A**–**C**, as indicated. *Denotes significant increase (p = 0.01) in comparison to basal conditions. **Denotes a significant increase (p = 0.01) in comparison to the control. **E** Comparison of the mean inward and outward conductances in 8 different patches in the conditions described in **A**–**C**, as indicated. Conductances were calculated from linear regressions of the corresponding I–V relationships. *Denotes significant increase (p = 0.01) in comparison to inward current control or to outward current control

To determine the direct effects of PTX on the TRPA1 current, we recorded ionic currents with inside-out configurations from SH-SY5Y cells, assessing the effect at − 60 mV. Before recording the currents, PTX (1 µM) was added to the extracellular solution followed by the addition of AITC, and the P_o_ and current–voltage relationships were derived. PTX significantly increased TRPA1 P_o_ and the inward and outward conductances (Fig. 3).

### Effects of PTX on TRPA1-Mediated Ca^2+^ Increase

**Fig. 4:**
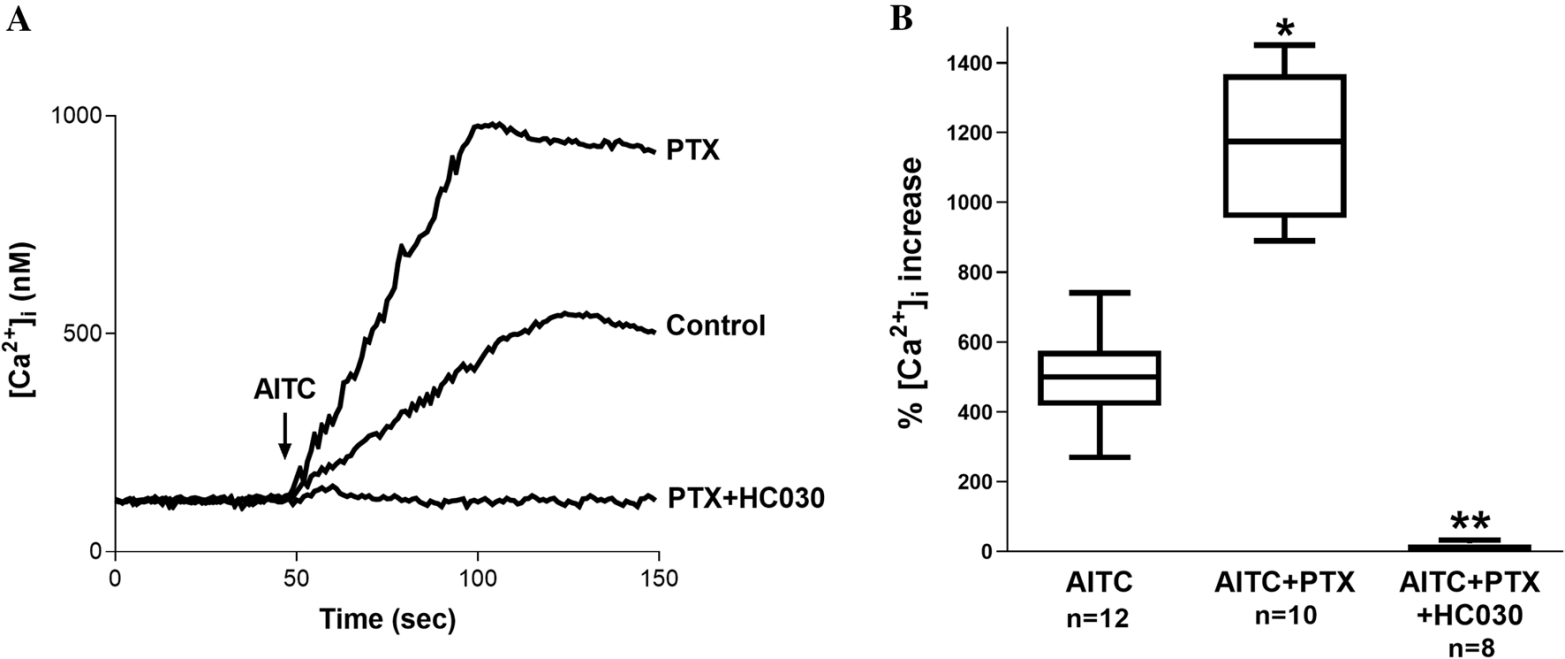
**A** Representative recordings of intracellular Ca^2+^ concentration in Fura-2-loaded SH-SY5Y cells under steady-state conditions and following AITC treatment in the presence of PTX and PTX + HC-030. Fluorescence was recorded for 150 s. The arrow indicates the moment in which AITC was added to the external solution. **B** Comparison between the mean maximal AITC-induced intracellular Ca^2+^ increase percentage in Fura-2-loaded SH-SY5Y cells, in the presence of PTX and PTX + HC-030. n is indicated in each case. *Denotes a significant decrease (p = 0.01) in comparison to the control

**Fig. 5:**
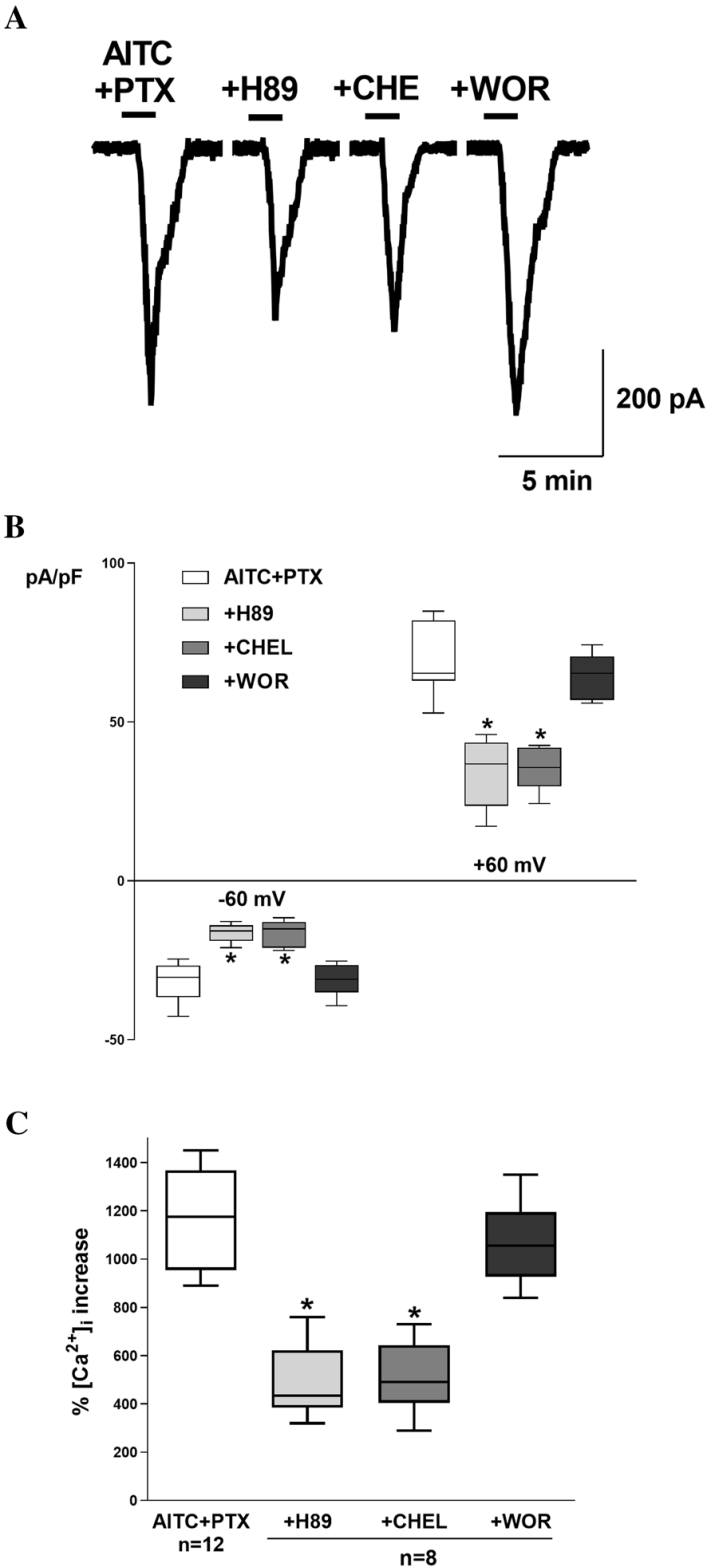
**A** Typical current (I) recording at − 60 mV in the presence of AITC + PTX and H89, chelerythrine (CHE) or wortmannin (WOR), as indicated. **B** Comparison between the mean maximal normalized current recorded at − 60 mV and + 60 mV under the same conditions described in **A**. n = 8 in all cases **C**. Comparison between the mean maximal intracellular Ca^2+^ increase percentage in Fura-2-loaded SH-SY5Y cells, in the presence of PTX + AITC under the same conditions described in **A** and **B**. n is indicated in each case. *Denotes a significant decrease (p = 0.01) in comparison to the control

We first measured changes in [Ca^2+^]_i_ following the AITC treatment of SH-SY5Y cells. AITC evoked a significant concentration-dependent increase in intracellular calcium in these cells (Fig. 4). To verify that the observed increase in [Ca^2+^]_i_ was mediated by TRPA1, we treated SH-SY5Y cells with AITC in the presence of HC-030. In these experiments, AITC completely failed to evoke an increase in [Ca^2+^]_i_, confirming TRPA1 as the pathway responsible for Ca^2+^. To test the hypothesis that PTX activates the TRPA1 channel, we evaluated the ability of PTX (1 µM) to evoke Ca^2+^ responses in cultured SH-SY5Y cells (Fig. 4). The AITC-induced Ca^2+^ increase was enhanced by PTX. Additionally, we explored the effect of kinase inhibition on the TRPA1- Ca^2+^ flux increased by PTX. The inhibition of PKA (10 µM H89) or PKC (10 µM chelerythrine) attenuated the effect of PTX on AITC-induced Ca^2+^, and the inhibition of PI3K (1 µM wortmannin) did not modify the PTX effects on TRPA1 (Fig. 5C).

## Discussion

The present study provides evidence for the expression and regulation of TRPA1 channels by PTX in SH-SY5Y cells. These effects may help to understand the underlying mechanism by which PTX induces neurotoxicity and subsequent neuropathy.

The expression of TRPA1 channels is mainly observed in sensory neurons from the DRG, as well as trigeminal and nodose ganglia [19]. In addition, TRPA1 channel expression in large cortical vessels [39], cerebral cortex [23], and hippocampus [40] has been reported. In the present study, we demonstrated the expression of TRPA1 channels at both the gene and protein levels in SH-SY5Y cells. Previously, TRPA1 activity has been reported in these cells [36], but TRPA1 expression was not evaluated.

The present study found that PTX increased [Ca^2+^]_i_ via TRPA1 activation in SH-SY5Y cells and that PTX increased TRPA1 expression. Therefore, these data suggested that TRPA1 expression and function are affected by PTX and interaction with PKA and PKC, and lead to propose complex calcium-mediated neurotoxicity with a convergence of kinase activation as the mechanism for TRPA1 modulation by phosphorylation [41].

In the PTX-induced peripheral neuropathy (PIPN) model, the TRPA1 channel is regulated by PKA and/or PKC phosphorylation through PAR2 cleavage and activation; the latter is mediated by the release of tryptase, which ends in CIPN and is characterized by mechanical, heat, and cold hypersensitivity [15]. In vivo studies have shown that inhibition of PKA activity reduces mechanical and thermal hyperalgesia induced by PTX [15, 42]. CIPN is associated with increased TRPA1 activity, a mechanism mediated by PKA. This regulatory mechanism of TRPA1 has been demonstrated in different models of neuropathic pain. For instance, endothelin 1 produces sensitization of TRPA1 channels via PKA activation in DRG neurons [43]. Furthermore, TRPA1 currents and membrane translocation are increased upon nociceptive signals in these cells, a process regulated by PKA and phospholipase C (PLC) signaling pathways [44, 45]. PKC is associated with mechanical hyperalgesia induced by PTX in an acute and chronic PIPN model [42]; and PKC is linked to neuropathic pain induced by vincristine [46]. Independently, G-protein coupled receptors (GPCRs), such as the B_1_ kinin receptor, has been shown to regulate TRPA1 in nociceptive transmission in the spinal cord, and inhibition of B_1_ decreases TRPA1 activity [42]. This effect has been demonstrated with other GPCRs of the bradykinin family (PAR2, PAR4, B_1,_ and B_2_) that involve the PLC and PKC signaling pathways. Apparently, TRPA1 serves as an integrator of inflammatory signaling secondary to the activation of GPCRs [47, 48]. PTX treatment involves the induction of oxidative stress, specifically the accumulation of H_2_O_2_ [36], thereby releasing and the CGRP sensory neuropeptide [19]. Furthermore, inhibition of TRPA1 channels reduced cold and mechanical allodynia induced by PTX [19], which suggests a strong interaction of PTX and kinase signaling, thus involving TRPA1 activity in the mechanisms of inflammatory pain [41, 49].

It is well known that PTX produces an alteration in [Ca^2+^]_i_ in central and peripheral models of neurotoxicity. For instance, PTX induces axonal degeneration by reducing the expression of the antiapoptotic protein Bcl-2, thereby altering inositol trisphosphate receptor (IP3R) activity, and intracellular Ca^2+^ homeostasis [50]. Furthermore, the microtubule stabilization effect induced by PTX prevents the association of stromal interaction molecule 1 (STIM1) with Orai1 and TRPC channels [51], which are essential mediators of store-operated Ca^2+^ entry (SOCE). Interestingly, TRPA1 channels prevent the STIM1/Orai1 association, leading to SOCE attenuation [52]. Apparently, SOCE alterations mediated by TRPA1 channels and/or microtubule stabilization are mechanisms involved in PTX-induced neurotoxicity.

PTX increases the binding capacity of neuronal Ca^2+^ sensor-1 (NCS-1) to IP3R, a Ca^2+^ channel expressed in the endoplasmic reticulum, inducing the release of Ca^2+^ from intracellular stores and increasing [Ca^2+^]_i_ which then activates calpain, a Ca^2+^-dependent protease [53, 54]. The activation of calpain leads to the degradation of NCS-1 and the subsequent decrease in intracellular Ca^2+^ [54, 55]. This initial increase in [Ca^2+^]_i_ can be further enhanced via TRP channels, such as TRPA1. Similar to DRG neurons [31, 56], we found that PTX treatment upregulated TRPA1 expression in SH SY5Y cells, as evidenced by WB and RT-PCR analyses. We hypothesized that PTX produces an increase in TRPV4 and TRPA1 channel expression and activity as a potentiating mechanism to maintain the [Ca^2+^]_i_ elevation previously generated by IP3R, and probably SOCE disruption, leading to Ca^2+^-dependent protease-induced cell death and neuronal damage. Further studies need to be conducted to test this hypothesis.

PTX increased TRPA1 currents and calcium influx in SH-SY5Y cells, as evidenced by conductance and P_o_ enhancement of the channel. Remarkably, these effects were exerted only in the presence of AITC, a specific TRPA1 agonist, indicating that the opening of the channel is required for the PTX effect. In addition, the chemotherapeutic agent modified TRPA1 permeability as demonstrated by the changes in the magnitude of conductance. These results demonstrated that the neurotoxic effects of PTX share similar characteristics on some TRP channels and the interaction among them needs to be further characterized. The present study demonstrated that PTX increased TRPA1 channel expression and activity in SH-SY5Y cells, which may support the role of TRPA1 channels in the mechanism involved in PTX-induced peripheral neurotoxicity. This evidence suggests a promising field to evaluate the clinical effectiveness of TRPA1 antagonism in preventing or treating PIPN. However, preclinical models and phase 1 studies involving TRPA1 antagonists alone or in combination with other TRPA1 modulators should be addressed to assess the feasibility of this approach. 

## Supplementary Information

Below is the link to the electronic supplementary material.

Supplementary file1 (TIF 139 kb)

Supplementary file2 (TIF 97 kb)

## Data Availability

The datasets generated during and/or analysed during the current study are not publicly available due to privacy policies of the Universidad Tecnológica de Pereira, but are available from the corresponding author on reasonable request.

## References

[1] Kudlowitz D, Muggia F (2013). Defining risks of taxane neuropathy: insights from randomized clinical trials. Clin Cancer Res.

[2] Gornstein EL, Schwarz TL (2017). Neurotoxic mechanisms of paclitaxel are local to the distal axon and independent of transport defects. Exp Neurol.

[3] Kober KM, Mazor M, Abrams G, Olshen A, Conley YP, Hammer M, Schumacher M, Chesney M, Smoot B, Mastick J (2018). Phenotypic characterization of paclitaxel-induced peripheral neuropathy in cancer survivors. J Pain Symptom Manag.

[4] Authier N, Gillet J-P, Fialip J, Eschalier A, Coudore F (2000). Description of a short-term Taxol®-induced nociceptive neuropathy in rats. Brain Res.

[5] Reeves BN, Dakhil SR, Sloan JA, Wolf SL, Burger KN, Kamal A, Le-Lindqwister NA, Soori GS, Jaslowski AJ, Kelaghan J (2012). Further data supporting that paclitaxel-associated acute pain syndrome is associated with development of peripheral neuropathy: North Central Cancer Treatment Group trial N08C1. Cancer.

[6] Loprinzi CL, Maddocks-Christianson K, Wolf SL, Rao RD, Dyck PJB, Mantyh P, Dyck PJ (2007). The paclitaxel acute pain syndrome: sensitization of nociceptors as the putative mechanism. Cancer J.

[7] Huehnchen P, Boehmerle W, Springer A, Freyer D, Endres M (2017). A novel preventive therapy for paclitaxel-induced cognitive deficits: preclinical evidence from C57BL/6 mice. Transl Psychiatry.

[8] Staff NP, Fehrenbacher JC, Caillaud M, Damaj MI, Segal RA, Rieger S (2020). Pathogenesis of paclitaxel-induced peripheral neuropathy: a current review of in vitro and in vivo findings using rodent and human model systems. Exp Neurol.

[9] Seretny M, Currie GL, Sena ES, Ramnarine S, Grant R, MacLeod MR, Colvin LA, Fallon M (2014). Incidence, prevalence, and predictors of chemotherapy-induced peripheral neuropathy: a systematic review and meta-analysis. PAIN®.

[10] Taillibert S, Le Rhun E, Chamberlain MC (2016). Chemotherapy-related neurotoxicity. Curr Neurol Neurosci Rep.

[11] Velasco R, Bruna J (2015). Taxane-induced peripheral neurotoxicity. Toxics.

[12] La Rovere RM, Roest G, Bultynck G, Parys JB (2016). Intracellular Ca(2+) signaling and Ca(2+) microdomains in the control of cell survival, apoptosis and autophagy. Cell Calcium.

[13] de Almeida AS, Rigo FK, De Prá SD-T, Milioli AM, Pereira GC, Lückemeyer DD, Antoniazzi CT, Kudsi SQ, Araújo DMPA, Oliveira SM (2020). Role of transient receptor potential ankyrin 1 (TRPA1) on nociception caused by a murine model of breast carcinoma. Pharmacol Res.

[14] Antoniazzi CTDD, Nassini R, Rigo FK, Milioli AM, Bellinaso F, Camponogara C, Silva CR, de Almeida AS, Rossato MF, De Logu F (2019). Transient receptor potential ankyrin 1 (TRPA1) plays a critical role in a mouse model of cancer pain. Int J Cancer.

[15] Chen Y, Yang C, Wang ZJ (2011). Proteinase-activated receptor 2 sensitizes transient receptor potential vanilloid 1, transient receptor potential vanilloid 4, and transient receptor potential ankyrin 1 in paclitaxel-induced neuropathic pain. Neuroscience.

[16] Materazzi S, Fusi C, Benemei S, Pedretti P, Patacchini R, Nilius B, Prenen J, Creminon C, Geppetti P, Nassini R (2012). TRPA1 and TRPV4 mediate paclitaxel-induced peripheral neuropathy in mice via a glutathione-sensitive mechanism. Pflügers Archiv-Eur J Physiol.

[17] Trevisan G, Materazzi S, Fusi C, Altomare A, Aldini G, Lodovici M, Patacchini R, Geppetti P, Nassini R (2013). Novel therapeutic strategy to prevent chemotherapy-induced persistent sensory neuropathy by TRPA1 blockade. Can Res.

[18] Meents JE, Ciotu CI, Fischer MJ (2019). TRPA1: a molecular view. J Neurophysiol.

[19] Raisinghani M, Zhong L, Jeffry JA, Bishnoi M, Pabbidi RM, Pimentel F, Cao D-S, Steven Evans M, Premkumar LS (2011). Activation characteristics of transient receptor potential ankyrin 1 and its role in nociception. Am J Physiol Cell Physiol.

[20] Kobayashi K, Fukuoka T, Obata K, Yamanaka H, Dai Y, Tokunaga A, Noguchi K (2005). Distinct expression of TRPM8, TRPA1, and TRPV1 mRNAs in rat primary afferent neurons with aδ/c-fibers and colocalization with trk receptors. J Comp Neurol.

[21] Story GM, Peier AM, Reeve AJ, Eid SR, Mosbacher J, Hricik TR, Earley TJ, Hergarden AC, Andersson DA, Hwang SW (2003). ANKTM1, a TRP-like channel expressed in nociceptive neurons, is activated by cold temperatures. Cell.

[22] Yamanaka M, Taniguchi W, Nishio N, Hashizume H, Yamada H, Yoshida M, Nakatsuka T (2015). In vivo patch-clamp analysis of the antinociceptive actions of TRPA1 activation in the spinal dorsal horn. Mol Pain.

[23] Kheradpezhouh E, Choy JM, Daria VR, Arabzadeh E (2017). TRPA1 expression and its functional activation in rodent cortex. Open Biol.

[24] Cordero-Morales JF, Gracheva EO, Julius D (2011). Cytoplasmic ankyrin repeats of transient receptor potential A1 (TRPA1) dictate sensitivity to thermal and chemical stimuli. Proc Natl Acad Sci.

[25] Talavera K, Startek JB, Alvarez-Collazo J, Boonen B, Alpizar YA, Sanchez A, Naert R, Nilius B (2020). Mammalian transient receptor potential TRPA1 channels: from structure to disease. Physiol Rev.

[26] Masuoka T, Kudo M, Yamashita Y, Yoshida J, Imaizumi N, Muramatsu I, Nishio M, Ishibashi T (2017). TRPA1 channels modify TRPV1-mediated current responses in dorsal root ganglion neurons. Front Physiol.

[27] Chen J, Joshi SK, DiDomenico S, Perner RJ, Mikusa JP, Gauvin DM, Segreti JA, Han P, Zhang X-F, Niforatos W (2011). Selective blockade of TRPA1 channel attenuates pathological pain without altering noxious cold sensation or body temperature regulation. Pain.

[28] Liu B, Escalera J, Balakrishna S, Fan L, Caceres AI, Robinson E, Sui A, McKay MC, McAlexander MA, Herrick CA (2013). TRPA1 controls inflammation and pruritogen responses in allergic contact dermatitis. FASEB J.

[29] Bautista DM, Jordt SE, Nikai T, Tsuruda PR, Read AJ, Poblete J, Yamoah EN, Basbaum AI, Julius D (2006). TRPA1 mediates the inflammatory actions of environmental irritants and proalgesic agents. Cell.

[30] Zhou S-Z, Zhou Y-L, Ji F, Li H-L, Lv H, Zhang Y, Xu H (2018). Analgesic effect of methane rich saline in a rat model of chronic inflammatory pain. Neurochem Res.

[31] Wu Z, Wang S, Wu I, Mata M, Fink DJ (2015). Activation of TLR-4 to produce tumour necrosis factor-alpha in neuropathic pain caused by paclitaxel. Eur J Pain.

[32] Li Y, Zhang H, Zhang H, Kosturakis AK, Jawad AB, Dougherty PM (2014). Toll-like receptor 4 signaling contributes to Paclitaxel-induced peripheral neuropathy. J Pain.

[33] Li Y, Adamek P, Zhang H, Tatsui CE, Rhines LD, Mrozkova P, Li Q, Kosturakis AK, Cassidy RM, Harrison DS (2015). The cancer chemotherapeutic paclitaxel increases human and rodent sensory neuron responses to TRPV1 by activation of TLR4. J Neurosci.

[34] Pittman SK, Gracias NG, Vasko MR, Fehrenbacher JC (2014). Paclitaxel alters the evoked release of calcitonin gene-related peptide from rat sensory neurons in culture. Exp Neurol.

[35] Nishio N, Taniguchi W, Sugimura Y, Takiguchi N, Yamanaka M, Kiyoyuki Y, Yamada H, Miyazaki N, Yoshida M, Nakatsuka T (2013). Reactive oxygen species enhance excitatory synaptic transmission in rat spinal dorsal horn neurons by activating TRPA1 and TRPV1 channels. Neuroscience.

[36] Özkal B, Övey İS (2020). Selenium enhances TRPA1 channel-mediated activity of temozolomide in SH-SY5Y neuroblastoma cells. Child's Nerv Syst.

[37] Payrits M, Borbely E, Godo S, Ernszt D, Kemeny A, Kardos J, Szoke E, Pinter E (2020). Genetic deletion of TRPA1 receptor attenuates amyloid beta- 1–42 (Abeta1-42)-induced neurotoxicity in the mouse basal forebrain in vivo. Mech Ageing Dev.

[38] Duan D-M, Wu S, Hsu L-A, Teng M-S, Lin J-F, Sun Y-C, Cheng C-F, Ko Y-L (2015). Associations between TRPV4 genotypes and body mass index in Taiwanese subjects. Mol Genet Genom.

[39] Sullivan MN, Gonzales AL, Pires PW, Bruhl A, Leo MD, Li W, Oulidi A, Boop FA, Feng Y, Jaggar JH (2015). Localized TRPA1 channel Ca2+ signals stimulated by reactive oxygen species promote cerebral artery dilation. Sci Signal.

[40] Bosson A, Paumier A, Boisseau S, Jacquier-Sarlin M, Buisson A, Albrieux M (2017). TRPA1 channels promote astrocytic Ca(2+) hyperactivity and synaptic dysfunction mediated by oligomeric forms of amyloid-beta peptide. Mol Neurodegener.

[41] Darby LM, Meng H, Fehrenbacher JC (2017). Paclitaxel inhibits the activity and membrane localization of PKCα and PKCβI/II to elicit a decrease in stimulated calcitonin gene-related peptide release from cultured sensory neurons. Mol Cell Neurosci.

[42] Dina O, Chen X, Reichling D, Levine J (2001). Role of protein kinase Cϵ and protein kinase A in a model of paclitaxel-induced painful peripheral neuropathy in the rat. Neuroscience.

[43] Zheng X, Tai Y, He D, Liu B, Wang C, Shao X, Jordt S-E, Liu B (2019). ETAR and protein kinase A pathway mediate ET-1 sensitization of TRPA1 channel: a molecular mechanism of ET-1-induced mechanical hyperalgesia. Mol Pain.

[44] Wang S, Dai Y, Fukuoka T, Yamanaka H, Kobayashi K, Obata K, Cui X, Tominaga M, Noguchi K (2008). Phospholipase C and protein kinase A mediate bradykinin sensitization of TRPA1: a molecular mechanism of inflammatory pain. Brain.

[45] Schmidt M, Dubin AE, Petrus MJ, Earley TJ, Patapoutian A (2009). Various signals involved in nociception regulate TRPA1 levels at the plasma membrane. Neuron.

[46] Joseph E, Levine J (2003). Sexual dimorphism for protein kinase Cε signaling in a rat model of vincristine-induced painful peripheral neuropathy. Neuroscience.

[47] Jonathan Y-X, Li L, Hasan R, Zhang X (2013). Excitation and modulation of TRPA1, TRPV1, and TRPM8 channel-expressing sensory neurons by the pruritogen chloroquine. J Biol Chem.

[48] Dai Y, Wang S, Tominaga M, Yamamoto S, Fukuoka T, Higashi T, Kobayashi K, Obata K, Yamanaka H, Noguchi K (2007). Sensitization of TRPA1 by PAR2 contributes to the sensation of inflammatory pain. J Clin Investig.

[49] Meotti FC, Figueiredo CP, Manjavachi M, Calixto JB (2017). The transient receptor potential ankyrin-1 mediates mechanical hyperalgesia induced by the activation of B1 receptor in mice. Biochem Pharmacol.

[50] Pease-Raissi SE, Pazyra-Murphy MF, Li Y, Wachter F, Fukuda Y, Fenstermacher SJ, Barclay LA, Bird GH, Walensky LD, Segal RA (2017). Paclitaxel reduces axonal Bclw to initiate IP3R1-dependent axon degeneration. Neuron.

[51] Galan C, Dionisio N, Smani T, Salido GM, Rosado JA (2011). The cytoskeleton plays a modulatory role in the association between STIM1 and the Ca2+ channel subunits Orai1 and TRPC1. Biochem Pharmacol.

[52] Albarrán L, Lopez JJ, Dionisio N, Smani T, Salido GM, Rosado JA (2013). Transient receptor potential ankyrin-1 (TRPA1) modulates store-operated Ca2+ entry by regulation of STIM1-Orai1 association. Biochim Biophys Acta Mol Cell Res.

[53] Boehmerle W, Splittgerber U, Lazarus MB, McKenzie KM, Johnston DG, Austin DJ, Ehrlich BE (2006). Paclitaxel induces calcium oscillations via an inositol 1,4,5-trisphosphate receptor and neuronal calcium sensor 1-dependent mechanism. Proc Natl Acad Sci USA.

[54] Boehmerle W, Zhang K, Sivula M, Heidrich FM, Lee Y, Jordt SE, Ehrlich BE (2007). Chronic exposure to paclitaxel diminishes phosphoinositide signaling by calpain-mediated neuronal calcium sensor-1 degradation. Proc Natl Acad Sci USA.

[55] Benbow JH, DeGray B, Ehrlich BE (2011). Protection of neuronal calcium sensor 1 protein in cells treated with paclitaxel. J Biol Chem.

[56] Matsumura Y, Yokoyama Y, Hirakawa H, Shigeto T, Futagami M, Mizunuma H (2014). The prophylactic effects of a traditional Japanese medicine, goshajinkigan, on paclitaxel-induced peripheral neuropathy and its mechanism of action. Mol Pain.

