# Supplementary figures and images for: Paclitaxel Regulates TRPA1 Function and Expression Through PKA and PKC
Source: Neurochem Res. 2022 Sep 13;48(1):295–304. doi: 10.1007/s11064-022-03748-0 (PMC9823074; doi:10.1007/s11064-022-03748-0)

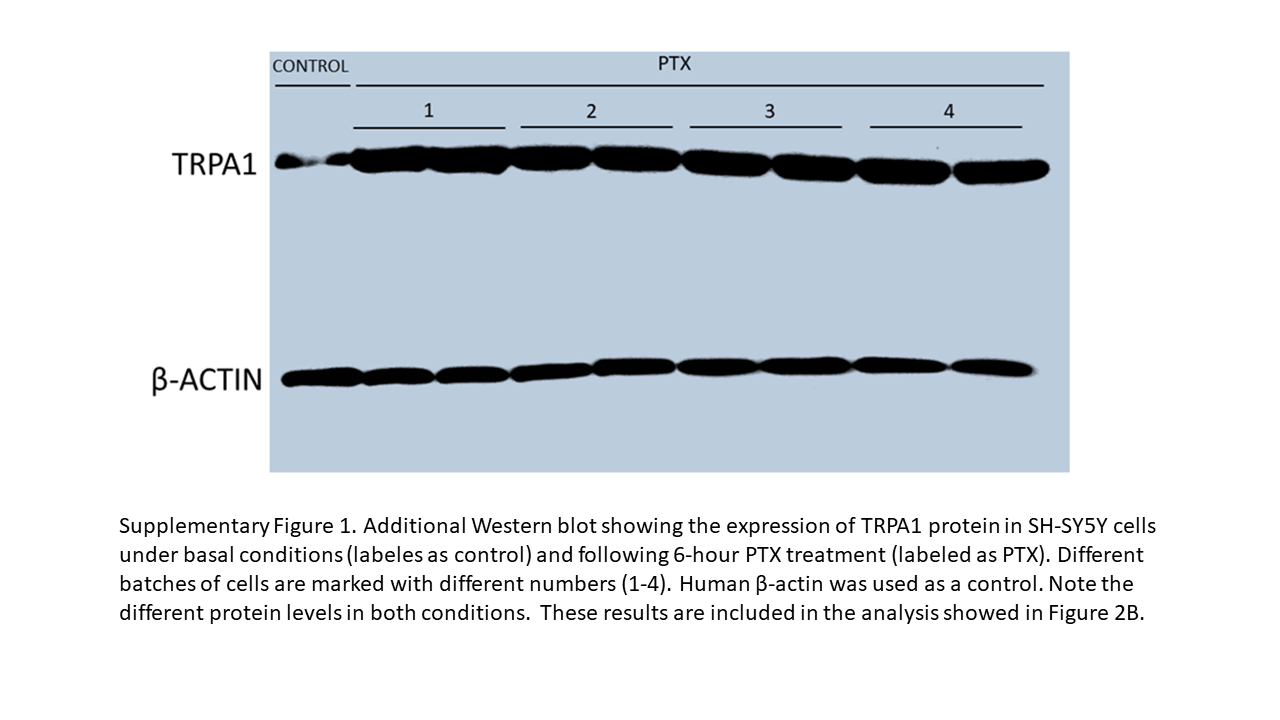

Supplement: Supplementary file 1 — Supplementary file1 (TIF 139 kb) [file 11064_2022_3748_MOESM1_ESM.tif]

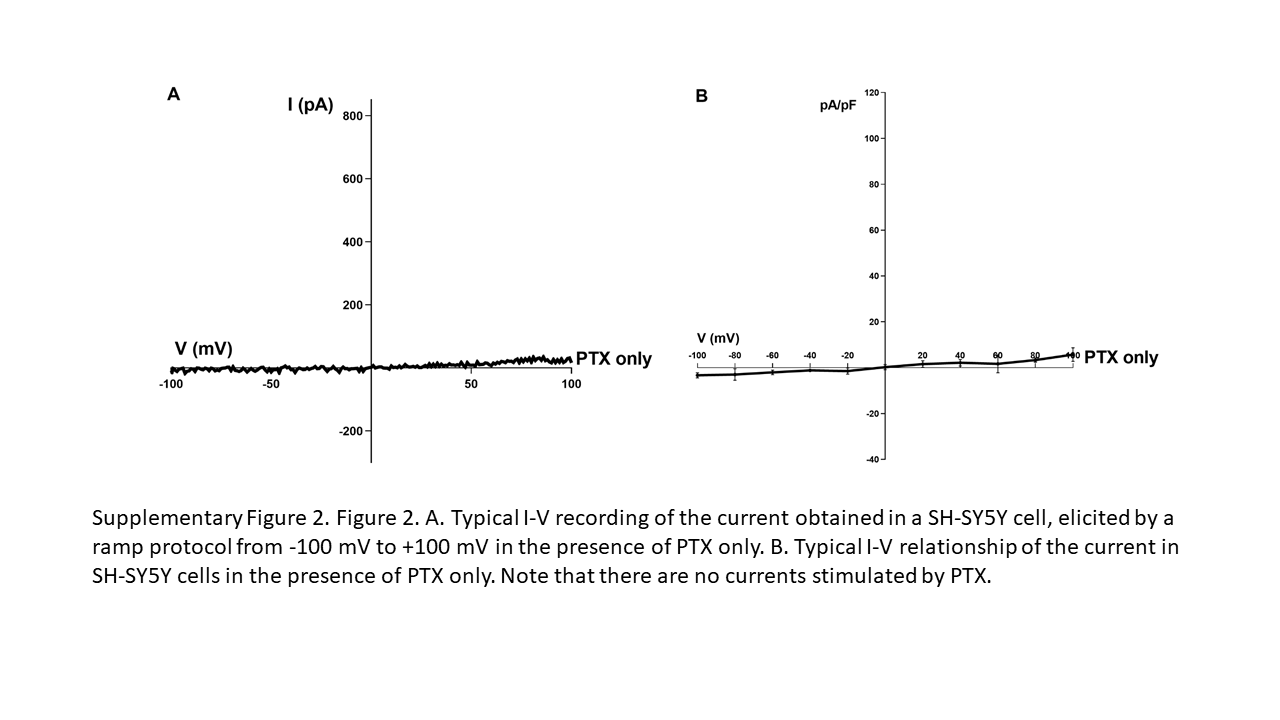

Supplement: Supplementary file 2 — Supplementary file2 (TIF 97 kb) [file 11064_2022_3748_MOESM2_ESM.tif]
